# The Role of Innate APOBEC3G and Adaptive AID Immune Responses in HLA-HIV/SIV Immunized SHIV Infected Macaques

**DOI:** 10.1371/journal.pone.0034433

**Published:** 2012-04-13

**Authors:** Yufei Wang, Trevor Whittall, Durdana Rahman, Evelien M. Bunnik, Robert Vaughan, Jørgen Schøller, Lesley A. Bergmeier, David Montefiori, Mahavir Singh, Hanneke Schuitemaker, Thomas Lehner

**Affiliations:** 1 Mucosal Immunology Unit, King's College London, Guy's Hospital, London, United Kingdom; 2 Department of Tissue Typing, King's College London, Guy's Hospital, London, United Kingdom; 3 AMC Medical Research, Amsterdam, The Netherlands; 4 Immudex, Copenhagen, Denmark; 5 Queen Mary, University of London, Barts and The London Schools of Medicine and Dentistry, Centre for Clinical and Diagnostic Oral Sciences, London, United Kingdom; 6 Department of Immunology, Duke University Medical Center, Durham, North Carolina, United States of America; 7 Lionex GmbH, Braunschweig, Germany; Imperial College London, United Kingdom

## Abstract

The AID/APOBEC family (activation induced deaminase/apolipoprotein B mRNA editing cytokine deaminase) in B cells play important roles in adaptive and innate immunity. Whereas APOBEC3G has been studied in CD4+ T cells and myeloid cells its functional potential in B cells has received little attention. AID combines two critical functions of antibodies, class switching and affinity maturation and may serve as a functional surrogate of protection. These functions were studied following systemic immunization of rhesus macaques with recombinant HLA constructs, linked with HIV and SIV antigens and HSP70 to dextran. The results showed significant upregulation of AID in CD20+ B cells, APOBEC 3G in CD27+ memory B cells and CD4+ effector memory T cells. After immunization the upregulated APOBEC 3G and AID were directly correlated in B cells (p<0.0001). Following challenge with SHIV SF162.P4 the viral load was inversely correlated with AID in B cells and APOBEC 3G in B and T cells, suggesting that both deaminases may have protective functions. Investigation of major interactions between DC, T cells and B cells showed significant increase in membrane associated IL-15 in DC and CD40L in CD4+ T cells. IL-15 binds the IL-15 receptor complex in CD4+ T and B cells, which may reactivate the DC, T and B cell interactions. The overall results are consistent with AID inhibiting pre-entry SHIV by eliciting IgG and IgA antibodies, whereas APOBEC 3G may contribute to the post-entry control of SHIV replication and cellular spread.

## Introduction

B cells do not express primary CD4 and CCR5 or CXCR4 coreceptors for HIV-1 binding and the virus does not replicate productively, unlike in CD4^+^ T cells. However, there is ample evidence that B cells can bind HIV-1 gp120 via surface Ig (VH3) [1], HIV-1 bound complement and its CR2 receptor (CD21) [2] or immune complexes of HIV-1 antibody with complement [3]. These surface-bound HIV-1 do not replicate unlike with DC-SIGN, also expressed by B cells, which may bind and internalize the virus and undergo low level replication [4]. These methods of HIV-1-bound B cells may result in trans infection of CD4^+^ T cells, though the mechanism of transmission has not been elucidated. Cell to cell contact between B cells and activated CD4^+^ T cells may be required, as has been suggested between follicular DC and CD4^+^ T cells in lymphoid tissue [5], [6].

B cells express two major deaminases, AID [7**]**–[****9] and APOBEC3G (A3G) [10**]**–[****13], which exert their functions by deaminating deoxycitidine to deoxyuridine. AID initiates somatic hypermutation (SHM), which generates high affinity antibodies by a process of affinity maturation [7**]**–[****9]. AID also elicits class switch recombination (CSR) of antibody isotypes from IgM to IgG, IgA and IgE [14]. A3G is an intracellular viral restricting factor, which induces lethal hypermutation or acts by a non-editing mechanism [10**]**–[****13]. Recent investigations have demonstrated that A3G is upregulated following mucosal immunization with SIV antigens and CCR5 peptides linked to the 70 kDa heat shock protein and is maintained for over 17 weeks [15]. The longevity of A3G mRNA and protein were associated with CD4^+^CCR5^+^ memory T cells in circulating PBMC, iliac lymph nodes and rectal cells of the immunized compared with unimmunized macaques. Furthermore, a significant increase in A3G mRNA in the CD4^+^CCR5^+^ circulating cells and the draining iliac lymph node cells was found following mucosal challenge with SIVmac251 in the immunized uninfected macaques, consistent with a protective effect exerted by A3G [15]. In another macaque study a combined mucosal adjuvant consisting of TLR agonists and IL-15, with peptides and boosted with MVA expressing SIV proteins also elicited long-lived A3G [16]. As with the previous investigation A3G expression was correlated with protection against rectal mucosal challenges with SIV mac251. Whereas A3G is an innate virus restricting factor, AID is involved mostly in adaptive immunity eliciting IgG and IgA antibody class switch and affinity maturation which may inhibit HIV and other retroviral infections.

These two deaminases do not seem to have been studied *in* vivo and we have explored their combined effects in HLA immunized macaques. Xenogeneic or allogeneic immunity is one of the most potent natural immune responses, MHC polymorphism plays a critical role in HIV control [17] and can elicit protection in immunized macaques [18**]**–[****22] and humans [23]. Furthermore, allo-immunization induces CD40L expression in CD4^+^ T cells [24] and may activate phosphorylation of IkB kinase complex, followed by nuclear translocation of NF-kB, which generates AID and induces CSR in B cells by binding to kB sites on IH promoters [25], [26]. CD40L bound to CD40 in DC activates ERK 1/2 and p38 MAP kinase and induces A3G expression [27]. Allogeneic stimulation *in vitro* and *in vivo* in humans also upregulates A3G mRNA in CD4^+^ T cells [28].

In this study immunization of rhesus macaques with HLA class I and II, trimeric HIV gp140, SIVp27, HSP70 and an adjuvant upregulated A3G in both CD4^+^ T cells and CD20^+^ B cells and the corresponding memory cells. AID was also upregulated in CD20^+^ B cells, which showed significant direct correlation with A3G in B cells. As both AID and A3G can be upregulated by immunization with the HLA constructs, we investigated their potential involvement in B and T cell protection against a SHIV challenge. An inverse correlation was recorded between the viral load and A3G, as well as AID in B cells in addition to A3G in CD4^+^ T cells. These findings are consistent with a dual function of immunization with the combined HLA-HIV/SIV vaccine, eliciting both innate and adaptive immunity, involving T and B cells and preventing or controlling SHIV replication and transmission.

## Results

### Immunization schedule and the effect on SHIV SF162.P4 challenge

Previous investigation of this series [29] demonstrated total prevention of SHIVSF162 infection in 2/8 macaques and significant decrease in viral load in the remaining 6 animals in group 3 (p<0.05), which were immunized with all vaccine components – recombinant HLA-class I and II, trimeric HIVgp140, SIVp27, HSP70 and the TiterMax adjuvant and challenged by IV SHIV SF162.P4. Macaques in the other three immunized groups had received all vaccine components, except SHIV in group 1, HLA I and II in group 2 and the adjuvant in group 4 (Table 1). All animals in the remaining immunized groups 1, 2 and 4, as well as the unimmunized group 5 were infected and showed no decrease in viral load. The 3 immunized groups had received all vaccine components, except for SHIV in group 1, HLA I and II in group 2 and the adjuvant in group 4 (Table 1).

**Table 1:** Vaccine constituents used for immunization in 4 groups of 8 rhesus macaques per group of rhesus macaques at 0, 4, 8 and 16 weeks administered by the SC route in all except group 4 IM, group 5 was unimmunized.

	Vaccine component					Adjuvant
Group	HLA class I	HLA class II	HIVgp 140	SIVp27	HSP70	Titer Max
1	+	+	−	−	+	+
2	−	−	+	+	+	+
3	+	+	+	+	+	+
4	+	+	+	+	+	−
5	−	−	−	−	−	−

Animals in group 1 consisted of 8 animals (except group 4 had 6 animals) challenged with SHIVSF162.P4 grown in C8166-CCR5^+^ T cells (HLA*A01, DR*04). All vaccine components were biotinylated, linked to streptavidin-bound dextran and formulated into an emulsion with the TiterMax adjuvant (except group 4 and 5).

### Upregulation of A3G expression in B cells

**Figure 1:**
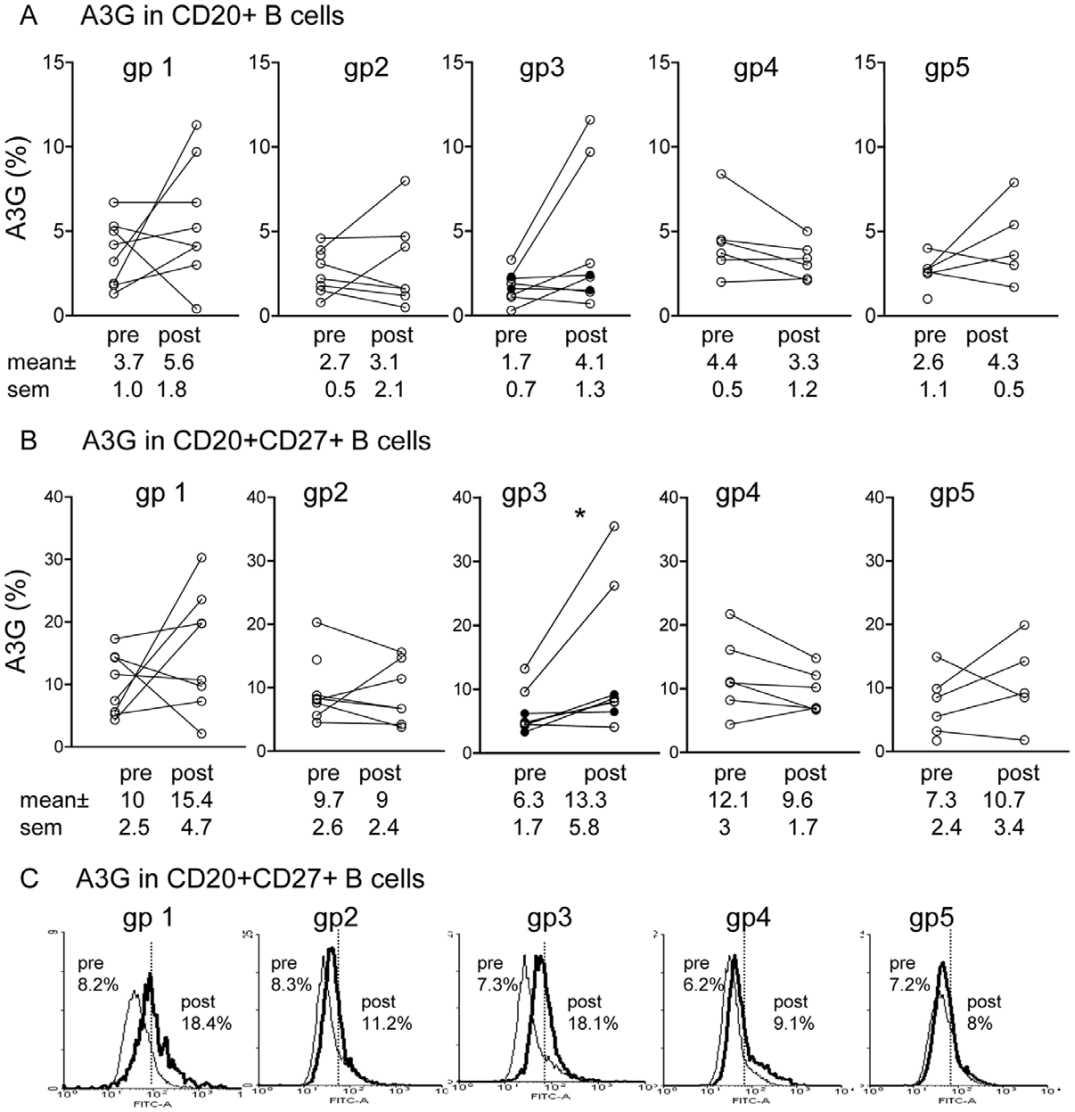
A3G in CD20^+^ and CD27^+^ memory B cells pre- and post-immunization in the 4 groups. Expression of A3G in (A) CD20^+^ B cells and (B) CD20^+^CD27^+^ memory B cells in 5 groups of macaques before and after the 4^th^ immunization assayed by flow cytometry with MAb to A3G, CD20 and CD27 and (C) representative illustration; (n = 8 per group, except gp4 n = 6). * p<0.05. In all figures the 2 uninfected macaques in group 3 are indicated by a solid circle.

**Figure 2:**
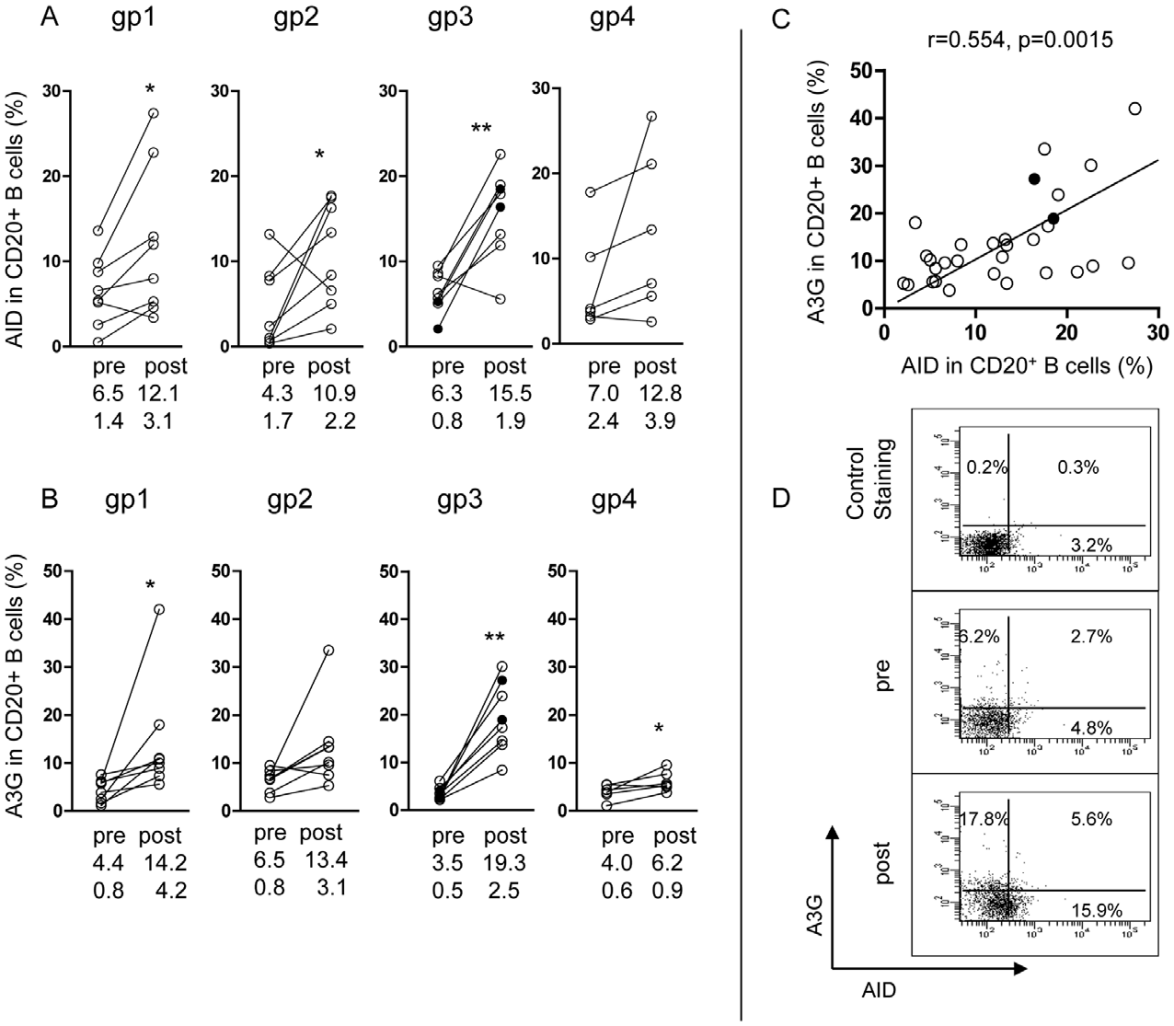
AID and A3G expression in CD20^+^ B cells pre- and post-2^nd^ immunization and their correlation. Comparative investigation of (A) AID and (B) A3G expression in CD20^+^ B cells before and after the 2^nd^ immunization in the 4 groups of immunized macaques; group 5 unimmunized controls remained unchanged (data not presented). Correlation between A3G and AID expression in CD20^+^ B cells in the 5 groups after 2^nd^ immunization is presented in (C). Representative flow cytometry of AID and A3G in pre- and post 2^nd^ immunization is shown in (D). * p<0.05 and ** p<0.01. In all figures the 2 uninfected macaques in group 3 are indicated by a solid circle.

The innate anti-viral factor A3G was studied in PBMC B cells of the 5 groups of macaques before and after the 4^th^ (final) immunization by flow cytometry. A3G in CD20^+^CD27^+^ memory B cells was increased significantly only in group 3 macaques (p<0.05; Fig****1B, C). Although A3G in CD20^+^ B cells was also upregulated, this reached significant levels after the 2^nd^ (Fig 2B,D) but not after the last immunization (Fig 1A). These results suggest that increased A3G expression was maintained only in CD20^+^CD27^+^ memory B cells and appears to be limited to group 3 immunized and protected macaques, which is consistent with previous long-term persistence of A3G in CD4^+^ memory T cells[15], [16].

### Upregulation of AID in B cells

To study the effect of immunization on the expression of AID in CD20^+^ B cells we examined first the baseline proportion of AID in B cells, which varied between 0.4**–**17.4% (mean±sem: 5.4±0.7%) in the macaques. An increase in AID was found after the first immunization, which reached significant levels after the second immunization in the 3 groups immunized with the adjuvant (Fig. 2A, C). Interestingly, group 1 immunized with HLA class I and II (but not HIVgp120) showed an increase in AID, the significance of which was higher (p = 0.025) than in group 2 immunized with HIV gp120 (but not HLA) (p = 0.043), however, group 3 immunized with both HLA and HIV antigens the significance increased further (p = 0.003), suggesting a partly additive function (Fig 2A, C). Both AID and A3G are produced in B cells, with a comparable pattern of responses to the 3 types of vaccines (groups 1, 2 and 3; Fig. 2A, B). Indeed, a very significant correlation was found between AID and A3G expression in CD20^+^ B cells in the combined immunized groups of macaques (p = 0.0015, Fig 2C) and a small proportion of B cells express both AID and A3G (Fig. 2D).

### A3G mRNA in PBMC and protein expression in CD4^+^ T cells

**Figure 3:**
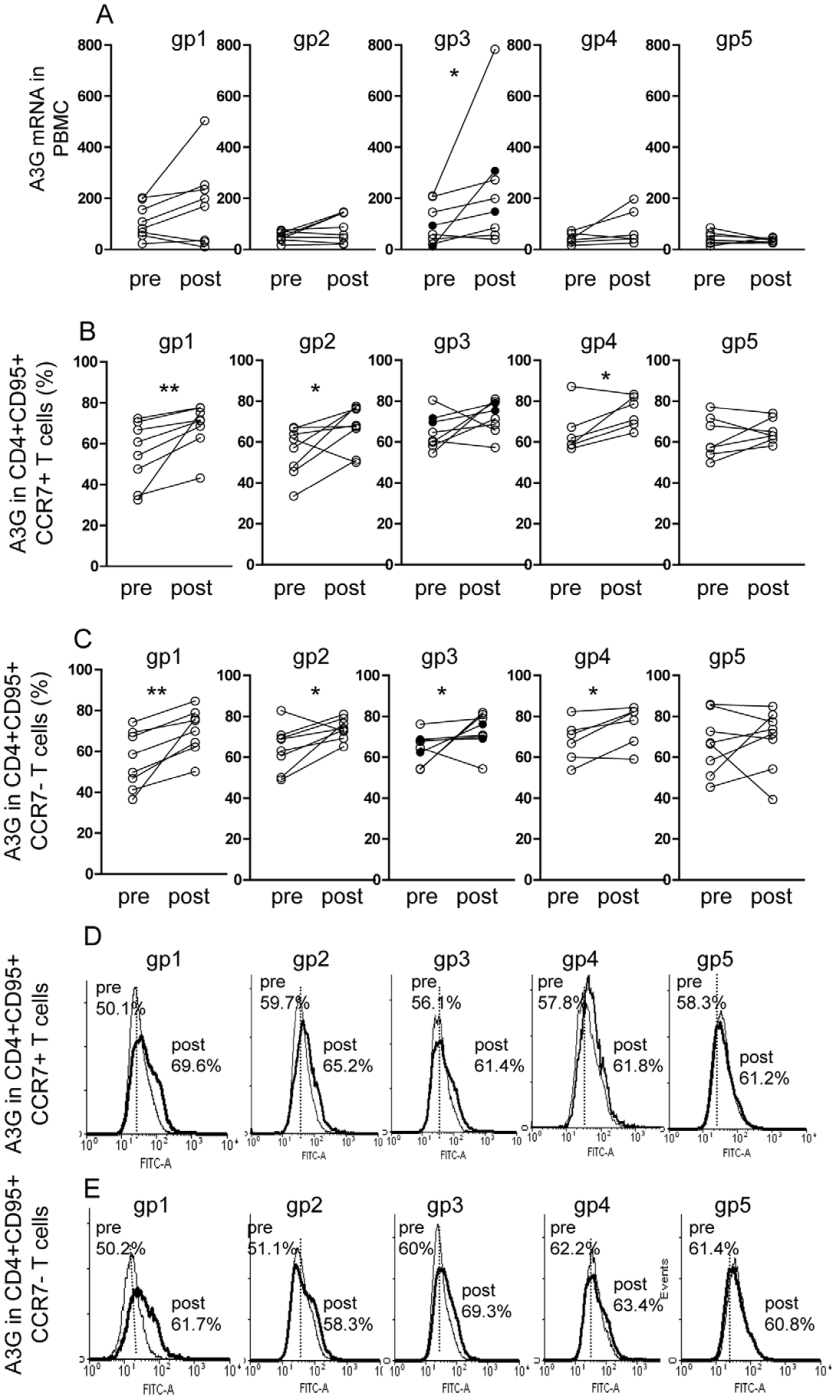
A3G mRNA in PBMC and A3G protein in central and effector memory CD4^+^ T cells. A3G mRNA expression in PBMC (A), protein in CD4^+^CD95^+^CCR7^+^ central (B) and CD4^+^CD95^+^CCR7^−^ effector memory T cell subsets (C) and representative illustration (D) and (E) respectively, in 5 groups of macaques before and after immunization, assayed by RT-PCR for A3GmRNA and flow cytometry using MAb to CD4, CD95 and CCR7. *p<0.05, **p<0.01. In all figures the 2 uninfected macaques in group 3 are indicated by a solid circle.

A significant increase in A3G mRNA assayed by RT-PCR was found in PBMC after the last immunization only in the protected group 3 animals (p = 0.046), none in group 2 and limited increase in groups 1 and 4 (Fig. 3A). The pre-immunization mRNA was 99(±28), which increased post-immunization over 2-fold to 236 (±85). A3G protein was then studied by flow cytometry in CD4^+^ memory T cells, which showed increased A3G expression in both CCR7^+^ central and CCR7^−^ effector memory T cells in all 4 immunized, except the former in group 3 macaques (Fig. 3B, C and representative profiles in D, E). It should be noted that in addition to A3G, A3F and to a lesser extent A3B are also capable of retroviral cDNA cytosine deamination [30] but these have not been studied due to limitations of PBMC required for the entire investigation.

### Comparative analysis of A3G expression between CD4^+^ CCR7^−^ effector memory T cells and CD20^+^CD27^+^ memory B cells

**Figure 4:**
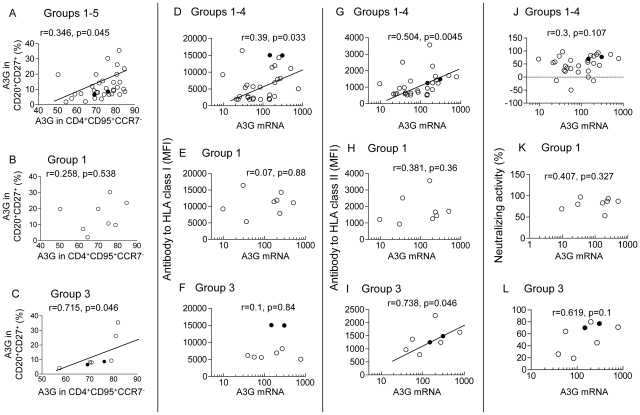
Correlation between A3G memory B and CD4^+^ T cells, and A3G mRNA with HLA or neutralizing antibodies. Correlation between A3G in CD20^+^CD27^+^ memory B cells and CD4^+^CD95^+^CCR7^−^ effector memory T cells (A) in all 5 groups, (B) in group 1 (without SHIV antigens) and (C) in group 3 macaques. Correlation between A3G mRNA in PBMC and serum anti-HLA class I antibodies (D**–**F), anti-HLA class II antibodies (MFI) (G**–**I) assayed by the Luminex HLA antibody method and neutralizing activity (J**–**L) determined by using a TZM-b1 assay in the corresponding groups. In all figures the 2 uninfected macaques in group 3 are indicated by a solid circle.

We explored the possibility that immunization with the HLA construct and SHIV may have elicited concomitant enhancement of A3G in CD4^+^ T and B cells. Indeed, A3G expression in CD4^+^ effector memory T cells (CD95^+^CCR7^−^) was directly correlated with CD20^+^CD27^+^ memory B cells (p = 0.045) in the whole cohort of animals (Fig. 4A). This result was replicated in the protected group 3 (p = 0.046), in contrast to the unprotected group 1 cohort (p = 0.537; Fig. 4C and B). These results are consistent with the concept that HLA immunization elicited parallel upregulation of A3G expression in subsets of memory CD4^+^ T cells and CD20^+^ B cells.

### Correlation between A3G mRNA in PBMC, A3G expression in CD20^+^ or CD27^+^ B cells and HLA, neutralizing antibodies

A potential relationship between A3G and anti-HLA or neutralizing antibodies was then explored, as HLA immunization may upregulate both A3G and AID deaminases in B cells. Significant direct correlation was found between A3G mRNA and both HLA-I (p = 0.033) and HLA-II antibodies (p = 0.004; Fig. 4D, G). Evaluation of the separate groups showed a significant direct correlation only in group 3 protected animals between A3G mRNA and anti-HLA II (DR) antibodies (p = 0.046; Fig. 4 I), and a trend of correlation between neutralizing activity and A3G mRNA, which failed to reach significance (r = 0.619; Fig. 4L).

### Correlation between AID expression in B cells and HLA-class I and II, and HIVgp120 antibodies

Expression of AID in CD20 B cells was also examined in relation to HLA class I and II antibodies. There was no correlation between AID expression in the immunized groups 1**–**4 and HLA-I or II antibodies (Fig. S1A, B). Examination of the separate groups, however showed a direct trend of correlation with HLA-II antibodies only in group 3 protected animals, which however does not reach significance (r = 0.61, p = 0.11; Fig. S1F). CSR is another functional activity of AID which was examined for HIVgp120 IgM, IgG and IgA antibodies. As expected only IgM to HIVgp120 was significantly upregulated after the first immunization, whereas IgG and IgA antibodies were upregulated only after the 4^th^ immunization (Fig. S2 A**–**F). Furthermore, direct correlation was observed between AID in CD20^+^ B cells, both with IgG and IgA antibodies to HIVgp120 in the combined groups (Fig. S2G, H), and high coefficient correlation (r = 0.62), though not significant between IgA and AID in group 3 (Fig. S2 I**–**J).

### Correlation between A3G expression in PBMC, CD4^+^ T cells, or AID in B cells and the viral load

**Figure 5:**
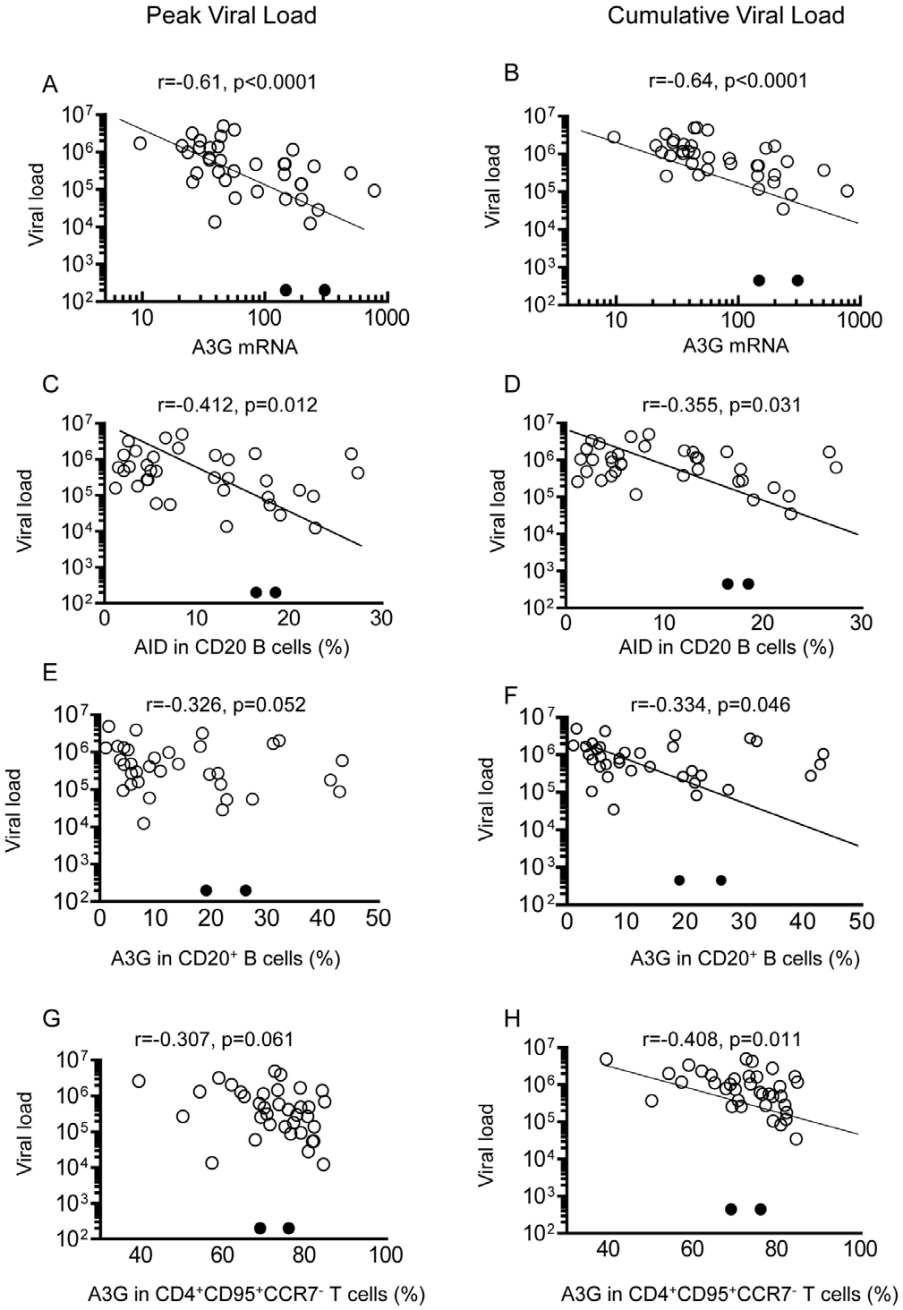
Correlation between the viral load and A3G mRNA, protein in B cells or AID. Correlation between PVL or CVL and A3G mRNA in PBMC (A,B), AID (C, D), A3G proteins in CD20^+^ B cells (E,F) and A3G in CD4^+^CD95^+^CCR7^−^ effector memory T cells (G,H). AID and A3G were assayed after the 4^th^ immunization, whereas CVL was calculated as the “area under the curve”. The two protected macaques are shown by open circles. Pearson's correlation coefficient was used for statistical analysis. In all figures the 2 uninfected macaques in group 3 are indicated by a solid circle.

We have then explored the critical question of correlates of protection following IV challenge with heterologous SHIVSF162.P4. A3G mRNA in PBMC showed very significant inverse correlation with the peak viral load (PVL, p<0.0001) and cumulative viral load (CVL, p<0.0001) in the entire series of animals (Fig. 5A, B). AID in CD20^+^ B cells also showed a significant inverse correlation both with PVL (p = 0.012) and CVL (p = 0.031) (Fig. 5C, D), as did A3G in CD20^+^ B cells with CVL (p = 0.046) and likely with PVL (p = 0.052) (Fig. 5E, F). A3G in CD20^+^ CD27^+^ memory B cells showed only a strong inverse trend with CVL (p = 0.07), but not with PVL (Fig. S3B, A). A3G protein in CD4^+^ T cells was also significantly inversely correlated between the effector memory CD4^+^ T cells (CD95^+^CCR7^−^) and CVL (p = 0.01, Fig. 5H) and to a lesser extent with the PVL (p = 0.061, Fig. 5G). However, the central memory CD4^+^ T cells (CD95^+^CCR7^+^) failed to show any correlation (Fig. S3C, D). Altogether, A3G in both CD4^+^ T cells and B cells and AID in B cells demonstrated significant inverse correlation with the viral load, suggesting that A3G and AID in these cells may contribute to inhibition of viral replication.

### Expression of IL-15 in DC and CD40L in CD4^+^ T cells

Allogeneic stimulation of CD4^+^ T cells in vitro induces CD40L [24], with significant increase in A3G mRNA [28]. Interaction between CD40L in CD4^+^ T cells and CD40 in DC activates transcription of membrane associated (ma) IL-15/IL-15Rα molecules in DC *in vitro* [*31*]. These observations lead us to examine maIL-15 on DC and CD40L expression on CD4^+^ T cells. Indeed, significant increase in malL-15 was found in group 3 (from 37.2%±3.5 to 45.1±4.8, p = 0.024), but not in the other 3 groups of macaques (Table 2A); representative flow cytometry illustration is presented in Fig. S5A. Examination of CD4^+^ T cell showed significant increases in CD40L expression only in group 3 macaques from 25.8(±3.6) to 44.7(±7.7) (p = 0.001) (Table 2B and Fig. S5B). These results confirm *in vivo* that immunization with the HLA constructs upregulates maIL-15 in DC and CD40L in CD4^+^ T cells of macaques in the protected group 3.

**Table 2:** Membrane-associated (ma) IL-15 of DC and CD40L expression of CD4^+^ T cells in 5 groups of macaques.

	(A) maIL-15 in DC				(B) CD40L in CD4^+^ T cells			
Group	Pre	Post	T	p	Pre	Post	t	p
1	34.3±5.2	40.5±4.5	1.222	0.131	29.6±(3.1)	32.1(5.0)	1.170	0.140
2	26.8±9	25.9±4.3	0.183	0.43	26.2(3.5)	25.3(2.5)	0.355	0.366
3	37.2±3.5	45.1±4.8	2.451	0.024	25.8(3.6)	44.4(7.7)	4.707	0.001
4	46.5±4.4	38.1±1.9	1.899	0.059	23.4(3.8)	19.3(3.6)	0.792	0.232
5	31.9±3.8	27.6±2.7	0.966	0.183	28.6(3.1)	24.9(3.9)	1.095	0.154
Anova		F = 3.999, p = 0.009				F = 48.761, p<0.0001		

(A) Membrane-associated (ma) IL-15 of DC and (B) CD40L expression on CD4^+^ T cells in 5 groups of macaques pre- and post-4^th^ immunization, presented as % mean (±sem). The significance between pre- and post-immunization was analysed by paired “t” test and differences between the 5 groups after immunization was analysed by ANOVA.

### Correlation between IL-15 and CD40L with A3G and AID expression

**Figure 6:**
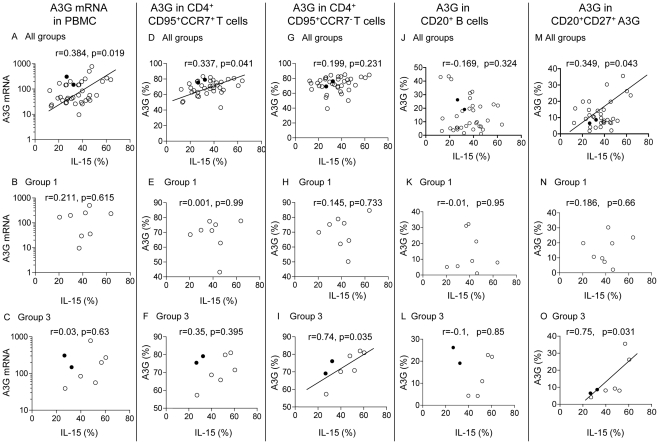
Correlation between DC maIL-15 and A3G mRNA or protein in CD4^+^ central and effector T or B memory cells. Correlation between maIL-15 on DC and A3G mRNA in PBMC (A**–**C), intracellular A3G protein in CD4^+^CD95^+^CCR7^+^ central memory cells (D**–**F), CD4^+^CD95^+^CCR7^−^ effector memory cells (G,I), CD20^+^ B cells (J**–**L) or CD20^+^CD27^+^ memory B cells (M**–**O) in the combined groups (1**–**5), group 1 or group 3 macaques, respectively. IL-15 and A3G were assayed after the last immunization and before the animals were challenged with SHIV SF162.P4. In all figures the 2 uninfected macaques in group 3 are indicated by a solid circle.

MaIL-15 was examined in DC of the 5 groups and showed significant direct correlation between IL-15 expression and A3G mRNA in PBMC (p = 0.019), and the central memory T cells (p = 0.041) at the time of challenge (Fig. 6A, D); these correlation were however, not reflected in groups 1 and 3 animals (Fig. 6 B**–**F). In contrast, IL-15 was correlated with A3G in the effector memory T cells in the protected group 3 (p = 0.035; Fig. 6) but not in the combined or group 1 animals (Fig. 6G, H). B cell analysis in all animals also demonstrated a significant correlation between maIL-15 of DC with A3G in CD20^+^CD27^+^ memory B cells (p = 0.043; Fig. 6M), which was also seen in group 3 macaques (p = 0.031; Fig. 6O), but not with A3G in CD20^+^ B cells.

Further analysis revealed significant direct correlation between CD40L in CD4^+^ T cells and A3G mRNA in PBMC (p = 0.034; Fig. S4A), but not with the CD4^+^ memory T cells in the 5 groups of animals (Fig. S4 B, C). Analysis of A3G and AID in CD20^+^ B cells failed to show any correlation with CD40L^+^CD4^+^ T cells (Fig. S4 D, F). However, A3G in CD20^+^ CD27^+^ memory B cells was significantly correlated with CD40L in CD4^+^ T cells (p = 0.02, Fig. S4E). Altogether, both maIL-15 in DC and CD40L in CD4^+^ T cells were directly correlated with A3G mRNA in PBMC, suggesting that immunization with the HLA-SHIV vaccine elicited the sequence of maIL-15→CD40L→A3G and this was most significant in A3G mRNA and A3G protein in CD20^+^ CD27^+^ memory B cells in the protected group 3 animals.

## Discussion

The major aims were to study the effect of immunization with novel HLA class I and II constructs, linked in proximity on dextran with trimeric HIV gp140, SIVgag p27 and HSP70, on A3G and AID expression in circulating B cells and A3G in CD4^+^ T cells. The potential dual effect of upregulating AID and A3G in B cells was explored by analyzing any association between them and those of A3G in CD4^+^ T cells and the 3 major antibody isotypes to the immunizing antigens. To enable evaluation of any protective effect of A3G and AID, the macaques were challenged with SHIV SF162.P4 and PVL and CVL were compared with A3G and AID expression between the immunized, unimmunized and control macaques lacking either HLA constructs or SHIV antigens.

Analysis of the entire cohort of 38 macaques clearly demonstrated that A3G mRNA in PBMC is inversely correlated with both the PVL and CVL (p<0.0001). Significant inverse correlation was found between the CVL and A3G protein in CD20^+^ B cells (p = 0.046) and the effector memory T cells (CD4^+^CD95^+^CCR7^−^, p = 0.01), but not CD4^+^ central memory T cells. These results suggest that A3G may exert a significant inhibitory effect on SHIV replication affecting the CVL and to a lesser extent the PVL. This appears to be quantitatively more significant in the CD4 effector memory T cells than CD20^+^CD27^+^ memory B cells. As B cells generally do not get infected by HIV or SHIV but they release readily A3G containing exosomes [32], the difference in expression of A3G between T and B cells may reflect the direct anti-viral function of A3G in T cells and an indirect effect exerted by B cells.

To evaluate A3G as a putative correlate of infection we analysed macaques in group 3, which were immunized with the whole vaccine (including HLA and HIV/SIV antigens, as well as the Titermax adjuvant), and was the only group to demonstrate either total protection or a significant decrease in viral load, as reported recently [29]. This required both the HLA constructs and SHIV antigens, as the previous studies with HLA immunization have clearly demonstrated that immunization without HLA or HIV/SIV antigens failed to elicit significant protection on challenge with SHIVSF162.P4 [29]. Analysis of the immune parameters in the 5 groups of macaques clearly indicate that animals mostly in the protected group 3 showed significant increase in A3G mRNA, though those in group1 (without HIV/SIV antigens) also showed a small increase in A3G mRNA, which was not significant. However, A3G in effector and central memory T cells was significantly upregulated in all 4 immunized groups of animals and the central memory T cells in groups 1, 2 and 4. In contrast CD20^+^CD27^+^ memory B cells were increased only in group 3. It was surprising that the CD27^+^ memory B cells appeared to be more discriminating than the CD4^+^ memory T cells, as the increase in A3G was confined to the protected group 3 animals. It is possible that the B cell contribution of A3G exosomes to CD4^+^ T cells and other SHIV-permissive cells may tip the balance in favour of A3G in countering the effect of vif. Furthermore, a significant direct correlation between A3G expressed in CD4^+^ effector memory T cells and CD27^+^ memory B cells was also found only in group 3 macaques. It is noteworthy that significant upregulation of A3G mRNA has been observed in group 3 animals already after the first immunization (p = 0.003). Altogether these results argue in favour of A3G produced both in T and B cells contributing to an innate protective immunity against SHIV infection.

The mechanism of the dual source of A3G is novel. For A3G produced by B cells to prevent SHIV infection it must be transmitted to CD4^+^ T cells, macrophages or DC. B cells are a major source of exosomes [32] in which A3G is a major component [33] which may confer virus restricted replication in CD4^+^ recipient cells [34]. Thus, a most likely mechanism is that A3G-rich exosomes from B cells will either directly or via nanotubes produced by B cells contact CD4 T cells [35]. Exosomes rich in A3G may have contributed to preventing SHIV infection in the passive transfer experiments [29], in which whole serum was used and AID may have affected the antibody class and affinity. Although B cells are not infected by HIV, an alternative mechanism is to bind the virions through CD21 complement binding receptor on B cells and transmit the virus to activated CD4^+^ T cells [2]. A3G produced by B cells might inhibit this process but this will need to be studied.

Nonetheless, immunization with the recombinant HLA constructs elicited only 25% prevention of SHIV infection, though the remaining animals showed a h decrease in viral load, compared with immunization with SIV inactivated whole CD4^+^ T cells, which induced up to 90% protection [18**]**–[****22]. These differences are likely to be accounted for by greater immunogenicity of whole cells with a multitude of antigen that included HLA A, B, C, DR, DQ and DP, compared with the pure recombinant HLA antigens. Furthermore, HLA of the immunizing CD4^+^ T cells/SIV was the same as the challenge SIV (grown in the same cells), unlike the immunizing recombinant HLA alleles, of which only 1 HLA class I and 1 class II (DR) allele were the same as those in the challenge SHIV. The dose of the candidate vaccine constituents was not optimized, as the priority was to demonstrate immunogenicity and evidence of protection.

As both A3G and AID deaminases are produced in B cells and HLA stimulation elicits both functions [28], [36], we explored the effect of HLA/SIV immunization on AID. A progressive increase in significance of AID expression was observed after the 2^nd^ immunization from group 2 with HIV/SIV antigens (p = 0.043), to group 1 with HLA antigens (p = 0.025) and group 3 with both HIV/SIV and HLA antigens (p = 0.003). A parallel increase with A3G was observed in the same samples of PBMC. This was consistent with finding a very significant correlation between A3G and AID in CD20^+^ B cells (p = 0.001), and the evidence that AID is a member of the APOBEC family [12], [37**]**–[****39]. Howevever, unlike A3G, AID appears to be restricted to the cytidine-deaminase dependent activity. Indeed, a direct correlation was found between A3G mRNA in B cells and anti-HLA–II antibodies in the protected group 3 animals (p = 0.046). Furthermore, AID in CD20^+^ B cells shows a trend that does not reach significance with HLA-class II IgG antibodies (p = 0.11) as do IgG and IgA antibodies to HIV gp120 (p = 0.10). These data suggest that upregulation of AID [40], which is maximal on immunization with the combined HLA-HIV/SIV vaccine candidate used in group 3, stimulated the adaptive function of antibodies. An alternative interpretation is based on the report that control of mouse Friend Virus 3 (Rfv3) infection is associated with murine A3 encoded by the Rfv3 gene, which influences control of the infection by neutralizing antibodies [41]. AID in contrast to its manifestations has not been studied in the context of immunization against micro-organisms, so it is noteworthy that AID showed a significant inverse correlation both with the peak and cumulative viral load.

Finally, we attempted to identify the cellular interactions between DC, CD4^+^ T cells and B cells and the mechanism of upregulation of A3G and AID. Activated CD4+ T cells express cell-surface CD40L, which bind CD40 on DC, stimulating the NF-κB transcription signaling pathway [31]. This activates membrane associated IL-15/IL15Rα molecules, which in turn bind the IL-15R complex on CD4^+^ T cells and reactivate the memory circuit [31]. A parallel memory circuit ligating CD40 molecules on B cells is likely to be involved, but we have not pursued it. CD40L-bound CD40 also activates ERK1/2 and p38 MAP kinase, inducing A3G mRNA, protein expression [27] and AID[36]. IL-15 upregulates directly A3G in CD4^+^ T cells by interacting with IL-15 receptor complex [28], which in turn upregulates CD40L in CD4^+^ T cells and activates CD40 molecules expressed by B cells. CD40L in the presence of HLA antibodies upregulate A3G and AID[36]. This is consistent with group 3 and 1, the only groups in which HLA antigens and the adjuvant were present, showing increase in maIL-15 DC and CD40L in CD4^+^ T cells. Upregulation of maIL-15 among other γ chain cytokines may also play a part in maintaining the homeostatic proliferation and conversion of naïve into memory T cells [42]. This concept has been recently highlighted by emphasis on IL-15 and IL-7 complementing weak triggering of the T cell antigen receptors [43].

As most HIV-1 infections are transmitted at mucosal surfaces (cervico-vaginal, rectal or penile), a dual protective immune function may be carried out by B cells, upregulating AID early after immunization, which activates CSR from IgM to IgG and IgA antibodies, and SHM inducing affinity maturation. Thus, the critical antibody functions expressed by AID might serve as a combined surrogate of protection, which has so far not been applied in vaccination studies. A3G elicits innate anti-viral activity and AID adaptive immune responses, which may exert post- and pre-entry anti-viral functions, respectively at the most vulnerable mucosal sites of infection. Overall the data are consistent with the hypothesis that HLA-SHIV immunization in macaques elicits both a conventional adaptive response, as demonstrated by HLA and HIV antibodies, and an innate A3G antiviral response. It is likely that A3G is upregulated early after an encounter with the virus and exerts a protective control over the virus, as the balance between A3G and vif will be shifted in favor of A3G. An alternative interpretation is that A3G might be of the LMM type, which is vif independent [10]. Furthermore, HSP70 was a component of the vaccine as it acts as a co-adjuvant and inhibits replication [44] possibly by binding Vif and A3G, thereby preventing Vif from ubiquitination and proteosomal degradation of A3G [45] or providing an A3G shield [12]. The innate response may control virus replication until an adequate antibody class, affinity and concentration develop and prevent or inhibits the infection. Antibodies may have been involved in anti-viral effect either by neutralizing antibodies, ADCC (antibody dependent cytotoxity [46], or by Fcγ receptor-mediated antibody dependent cellular virus inhibition [47]. The critical contribution of antibodies in protection elicited by the vaccine construct has been presented [29]. It should be noted however, that A3G may under some experimental conditions and especially with low deaminase activity promote mutation of the virus, which could affect its virulence [48], [49]. This possibility is unlikely in the present study, as one of the 4 immunization groups were protected and the other 3 did not show an increase in viral load.

The mechanism of AID function in SHIV restriction is dependent on its two constitutive functions, CSR and SHM. The IgM-IgG-IgA switch has been demonstrated with HIVgp120 antibodies, as only significant IgM class of antibodies were found after the 1^st^ and only IgG and IgA antibodies after the last immunization, consistent with class switch recombination. SHM is inferred by the specific antibodies to both HLA-class I and II and HIVgp120. Although the antibody titres were high, affinity was not tested, so we are unable to say if affinity maturation has also been elicited by SHM. Altogether, mostly IgG antibodies in the systemic circulation and IgA as well as IgG antibodies in the mucosal tissues will have exerted anti-viral effect. Further work is needed to ascertain whether AID may function as a composite marker of the level of antibody class, titre and affinity and whether testing for AID is of greater significance than the sum of CSR and SHM in the antiviral function.

The innate immune responses should contribute a significant novel dimension to the known advantages of alloimmunization in advancing a protective vaccine. The exposure to HLA class I or II antigen elicits a rapid primary antibody response, which engages the virus and is independent of viral mutation that may subsequently take place. However, as both HLA and HIV antigens were essential in eliciting protection with the recombinant antigens [29], the contribution that each makes to the protective mechanism will need to be elucidated. We suggest that the early innate A3G anti-viral effect, combined with AID enhanced IgG and IgA anti-HLA and SHIV antibody responses, offers an alternative preventative immunization strategy against HIV infection.

## Materials and Methods

### Ethics Statement

The study was carried out in compliance with the provisions and general guidelines of the Swedish Animal Welfare Agency, and all procedures were approved by the Ethical Committee on Animal Experiments of North Stockholm (permit number N90/06). Thirty-eight female rhesus macaques (*Macaca mulatta*) of Chinese origin, 3**–**5 years old at the start of the study, were housed in the Astrid Fagraeus laboratory at the Swedish Institute for Infectious Disease Control. Housing and care procedures were in compliance with the provisions and general guidelines of the Swedish Animal Welfare Agency all procedures were approved by the Local Ethical Committee on Animal Experiments. Immunizations and blood sampling were performed under sedation with ketamine 10 mg/kg intramuscularly (i.m.; Ketaminol 100 mg/ml, Intervet, Sweden). Before entering the study, all animals were confirmed to be negative for simian immunodeficiency virus (SIV), simian T-cell lymphotropic virus and simian retrovirus type D.

The serum from a healthy AB^+^ blood donor as a source of complement was acquired from Amsterdam, The Netherlands Blood Bank.

### Vaccine preparation

Four HLA class I alleles with the appropriate peptides were selected: (1) HLA-A*01:01(IVDCLTEMY), (2) HLAA*02:01(GLIQLVEGV), (3) HLA-A*03:01(RIAAWMATY), (4) HLA-A*11:01(VTDF SVIK) and one HLA class II allele HLA DRB1*04:01; these will cover >90% of a Caucasian population. The biotinylated vaccine components peptide-MHC class I and class II complexes, trimeric HIVgp140, SIVp27 and HSP70_359-609_ were linked to streptavidin coated divinyl sulfone acid activated dextran backbone [50].

### Immunization and SHIV challenge of 5 groups of macaques

The vaccines and immunization schedule has been reported elsewhere [29]. Briefly, 4 groups of 8 and 1 group of 6 Chinese rhesus macaques were immunized SC at 0, 4, 8 and 16 weeks: group 1 animals received recombinant HLA class I and II, HSP70 and TiterMax adjuvant, group 2 had HIV gp120, SIV p27, HSP70 and TiterMax group 3 had all vaccine components, HLA class I and II, HIVgp120, SIVp27, HSP70 and TiterMax, group 4 had the same as group 3, but without the adjuvant (n = 6) and group 5 was unimmunized (Table 1). The animals were challenged IV with 18 MID50 of SHIVSF162P4 (kindly provided by Nancy Miller at NIAID, NIH), propagated in the human T cell line C8166-CCR5, which expresses HLA-A*01 and –DRB1*04. Viral load was monitored as reverse transcriptase activity in plasma using the 26 ExaVir® Load version 3 kit (Cavidi Tech AB, Uppsala, Sweden) and translated to RNA equivalents/ml.

### Real-time PCR for APOBEC3G mRNA in PBMC

Macaque PBMC (1×10^6^) were thawed from cryo-perserved samples into RPMI 1640 medium supplemented with 10% FCS. After centrifugation at 500 g for 5 min the cell pellets were washed with PBS. RNA was isolated using a Total RNA Isolation Kit (Promega, Southampton,UK), quantified by spectrophotometry (GeneQuant II, Pharmacia Biotech), and cDNA was generated from RNA by using the Reverse Transcription System (Promega), according to the manufacturer's instructions. Relative amount of A3G mRNA was quantified by real-time PCR (ABI Prism 5700) using the Platinum SYBR green qPCR SuperMIX-UDG with ROX (Invitrogen Life Technologies) as described before [15]. The results were expressed as the copy number per ng of total RNA. mRNA studies on isolated B and T cells were not possible, as the yield of those cells was inadequate with the available number of PBMC.

### A3G and AID Protein Studies by Flow Cytometry

Intracellular A3G protein expression in CD4^+^ T cells, A3G and AID in CD20^+^ B and the corresponding memory cells were assayed by intracellular staining with anti-A3G Mab (ImmunoDiagnostics Inc, Woburn MA) and rabbit anti-AID Ab (Abnova, Caltagmedsystems, UK) in combination with cell surface staining. The pre- and post-immunized samples were analysed in parallel in each assay. The viability of thawed cells was checked by trypan blue exclusion and was greater than 85%. Macaque CD4^+^ naïve cells were identified by CD95 low and memory cells by CD95 high expression with antibodies to CD4 and CD95 (BD Biosciences, Oxford). Central memory cells were identified as CCR7^+^ and effector memory cells as CCR7^−^ cells with anti-CCR7 antibodies (R&D System, Oxford, UK), as described before [15]. B cells were identified by antibodies to CD20 and memory B cells by CD27 (BD Biosciences, Oxford). After cell surface staining, the cells were washed and fixed lightly with a fixation buffer containing formaldehyde for 3 mins (eBioscience, Insight Biotechnology, London UK), followed by treatment with the permeabilization buffer (eBioscience). FITC labeled A3G mAb and rabbit anti-AID were added to the samples followed by APC labeled sheep anti-rabbit secondary antibody (ABDserotec Oxford) at 1∶100 dilution. The cells were analysed by flow cytometry on FACSCanto II (BD Biosciences), using FACS Diva software. The pre-immunization data in Fig. 1 and 2 are strictly not comparable, as those in Fig. 1 were stained for CD4 and A3G, whereas those in Fig. 2 were stained not only for CD4 and A3G but also AID, The reproducibility of AID and A3G assays in B cells were carried out on 6 different samples of PBMC and repeated measures of ANOVA showed no significant difference of either A3G (F = 0.23, p = 0.8) or AID (F = 0.28, p = 0.78).

### Flow cytometry analysis of IL-15 expression in DC and CD40L expression in CD4+ T cells

Macaque DC were identified by incubating 1×10^6^ PBMC with a cocktail of antibodies, showing high expression of HLA class II and negative for CD14, CD20, CD3 and CD56 (BD Biosciences, UK). IL-15 expression in the DC population was then analysed with PE labeled anti-IL-1 Mab (R&D Systems, Oxford, UK). For CD40L staining, 5 μl of FITC-conjugated mAb to CD40L or isotype control antibody (BD Biosciences, BD Europe) was added to 2x10^5^ PBMC in 50 μl medium and were incubated for 5 hours. After washing the cells were stained for CD4 and analysed by flow cytometry.

### Assays of serum antibodies to HLA class I and II and HIV gp120

Serum HLA class I and class II antibodies were assayed using the Luminex Labscreen mixed HLA antibody method (One Lambda Inc., Canoga Park, CA). Labscreen single antigen beads (One Lambda Inc.) was used to show the HLA antigen specificity. The assays were carried out according to the manufacturer's instructions. Anti-HLA class I antibodies were assayed against HLA A0101, A0201, A0301, A1101 and A2402 and anti HLA class II antibody against DR0401 as described before [29].

Serum IgG, IgA and IgM antibodies to HIVgp120 were assayed by ELISA (enzyme-linked immunosorbent assay) as described previously (Yang et al., in preparation). Briefly, plates were coated with a pre-determined optimal concentration of HIVgp120 (1 µg/ml, NIBSC, Potters Bar, UK) and were then incubated with double dilution of serum (starting dilution of 1:100). Bound antibody was detected by incubation with rabbit IgG anti-monkey IgA (8 µg/ml) (Nordic Immunological Laboratories, Tilberg, The Netherlands), IgM or IgG antibodies (2 μg/ml; Sigma-Aldrich, Poole, Dorset, UK), followed by affinity-purified goat anti-rabbit IgG-alkaline phosphatase conjugate (Sigma). Antibody titres were presented as O.D by calculating the area under curve for each serum titration curve.

### Neutralizing activity

Serum HIV neutralization activity was tested for inhibition of SHIV-SF162.P4, replication in C8166-CCR5 cells (SHIV-SF162.P4), using a TZM-bl based assay, as described previously [29]. Neutralization activity in serum was analyzed both in the presence of complement, using serum from a healthy AB+ blood donor as a source of complement, and in the absence of complement, using heat-inactivated AB+ serum. Briefly, SHIV SF162.P4 was incubated in serial dilutions of macaque serum and added to TZM-bl cells, luminescence was measured and the percent neutralization was calculated by determining the reduction in luciferase expression in the presence of neutralizing agent compared to the cultures with virus only. Fifty % inhibitory dilutions (ID_50_) were determined by linear regression.

### Statistical analysis

All results are expressed as mean (±sem). The paired Student's t test was used for analysis of significance between pre- and post-immunized animals. Spearman rank or Pearson correlation coefficient was applied for analysis of correlation. The total viral load was calculated as the “area under the curve”. The total anti-HLA class I antibodies was calculated by adding up the MFI values of 5 HLA class I antigen specific antibodies. The antibody levels were presented as total OD values of each serum titre, expressed as area under the curve [51]. Probability value (p) <0.05 was considered to be significant.

## Supporting Information

Figure S1 **Correlation between AID and HLA-class I antibodies in groups 1–4, 1 and 3.** Correlation between AID in CD20^+^ B cells and anti-HLA class I (A,C,E) or anti-HLA class II antibodies (B, D, F); (A,B) in the immunized groups 1**–**4, (C,D) in group 1 and (E,F) in group 3 macaques. In all figures the 2 uninfected macaques in group 3 are indicated by a solid circle.
(TIF)

Figure S2 **Comparison of HIVgp120 antibodies and correlation with AID in B cells.** Comparison of serum HIV gp120 specific IgM, IgG and IgA antibodies, in the 5 groups of macaques after the 1^st^ (A**–**C) and 4^th^ (D**–**F) immunization; correlation between AID in CD20+ B cells and HIVgp120 IgG (G) or IgA (H) antibodies in all groups and in group 3 macaques (I,J). The antibodies were measured by ELISA and expressed as mean (±sem) of the OD (area under the curve). *p<0.05, **p<0.01 and ***p<0.001 compared with the untreated group 5 controls. In all figures the 2 uninfected macaques in group 3 are indicated by a solid circle.
(TIF)

Figure S3 **Indirect trend of correlation between the viral load and A3G in memory B and T cells.** Indirect trend of correlation between the peak or cumulative viral load and A3G in (A,B) CD20^+^CD27^+^ memory B cells and (C,D) CD4^+^CD95^+^CCR7^+^ memory T cells. In all figures the 2 uninfected macaques in group 3 are indicated by a solid circle.
(TIF)

Figure S4 **Correlation between CD40L and A3G mRNA in PBMC or A3G in memory B and central T cells**. Correlation between CD4^+^ CD40L^+^ T cells and (A) A3G mRNA in PBMC, (B) A3G protein in CD4^+^CD95^+^CCR7^+^ central, (C) CD4^+^CD95^+^CCR7^-^ effector memory T cells, (D) CD20^+^ B cells and (E) CD20^+^CD27^+^ memory B cells. (F) Correlation between CD40L and AID in CD20^+^ B cells in the 5 groups of animals. In all figures the 2 uninfected macaques in group 3 are indicated by a solid circle.
(TIF)

Figure S5 **Rrepresentative flow cytometry of maIL-15 DC and CD40L expression of CD4^+^ T cells.** Representative flow cytometry illustrations are presented (A) for maIL-15 and (B) CD40L; pre- (thin line) and post-immunization (bold line). In all figures the 2 uninfected macaques in group 3 are indicated by a solid circle.
(PPT)

## References

[1] Berberian L, Goodglick L, Kipps TJ, Braun J (1993). Immunoglobulin VH3 gene products: natural ligands for HIV gp120.. Science.

[2] Moir S, Malaspina A, Li Y, Chun TW, Lowe T (2000). B cells of HIV-1-infected patients bind virions through CD21-complement interactions and transmit infectious virus to activated T cells.. J Exp Med.

[3] Jakubik JJ, Saifuddin M, Takefman DM, Spear GT (2000). Immune complexes containing human immunodeficiency virus type 1 primary isolates bind to lymphoid tissue B lymphocytes and are infectious for T lymphocytes.. J Virol.

[4] Rappocciolo G, Piazza P, Fuller CL, Reinhart TA, Watkins SC (2006). DC-SIGN on B lymphocytes is required for transmission of HIV-1 to T lymphocytes.. PLoS Pathog.

[5] Heath SL, Tew JG, Szakal AK, Burton GF (1995). Follicular dendritic cells and human immunodeficiency virus infectivity.. Nature.

[6] Joling P, Bakker LJ, Van Strijp JA, Meerloo T, de Graaf L (1993). Binding of human immunodeficiency virus type-1 to follicular dendritic cells in vitro is complement dependent.. J Immunol.

[7] Muramatsu M, Kinoshita K, Fagarasan S, Yamada S, Shinkai Y (2000). Class switch recombination and hypermutation require activation-induced cytidine deaminase (AID), a potential RNA editing enzyme.. Cell.

[8] Peled JU, Kuang FL, Iglesias-Ussel MD, Roa S, Kalis SL (2008). The biochemistry of somatic hypermutation.. Annu Rev Immunol.

[9] Stavnezer J, Guikema JE, Schrader CE (2008). Mechanism and regulation of class switch recombination.. Annu Rev Immunol.

[10] Chiu YL, Greene WC (2006). Multifaceted antiviral actions of APOBEC3 cytidine deaminases.. Trends Immunol.

[11] Conticello SG, Ganesh K, Xue K, Lu M, Rada C (2008). Interaction between antibody-diversification enzyme AID and spliceosome-associated factor CTNNBL1.. Mol Cell.

[12] Harris RS, Bishop KN, Sheehy AM, Craig HM, Petersen-Mahrt SK (2003). DNA deamination mediates innate immunity to retroviral infection.. Cell.

[13] Sheehy AM, Gaddis NC, Choi JD, Malim MH (2002). Isolation of a human gene that inhibits HIV-1 infection and is suppressed by the viral Vif protein.. Nature.

[14] Di Noia JM, Williams GT, Chan DT, Buerstedde JM, Baldwin GS (2007). Dependence of antibody gene diversification on uracil excision.. J Exp Med.

[15] Wang Y, Bergmeier LA, Stebbings R, Seidl T, Whittall T (2009). Mucosal immunization in macaques upregulates the innate APOBEC 3G anti-viral factor in CD4(+) memory T cells.. Vaccine.

[16] Sui Y, Zhu Q, Gagnon S, Dzutsev A, Terabe M (2010). Innate and adaptive immune correlates of vaccine and adjuvant-induced control of mucosal transmission of SIV in macaques.. Proc Natl Acad Sci U S A.

[17] Study TIHC (2010). The major genetic determinants of HIV-1 control affect HLA class I peptide presentation.. Science.

[18] Desrosiers RC, Wyand MS, Kodama T, Ringler DJ, Arthur LO (1989). Vaccine protection against simian immunodeficiency virus infection.. Proc Natl Acad Sci U S A.

[19] Murphey-Corb M, Martin LN, Davison-Fairburn B, Montelaro RC, Miller M (1989). A formalin-inactivated whole SIV vaccine confers protection in macaques.. Science.

[20] Stott EJ, Chan WL, Mills KH, Page M, Taffs F (1990). Preliminary report: protection of cynomolgus macaques against simian immunodeficiency virus by fixed infected-cell vaccine.. Lancet.

[21] Arthur LO, Bess JW, Urban RG, Strominger JL, Morton WR (1995). Macaques immunized with HLA-DR are protected from challenge with simian immunodeficiency virus.. J Virol.

[22] Hunsmann G (1995). Protection of macaques against simian immunodeficiency virus infection with activated vaccines. Comparison of adjuvants, doses and challenge viruses.. Vaccine.

[23] Wang Y, Tao L, Mitchell E, Bravery C, Berlingieri P (1999). Allo-immunization elicits CD8+ T cell-derived chemokines, HIV suppressor factors and resistance to HIV infection in women.. Nat Med.

[24] Bartlett A, McCall J, Ameratunga R, Munn S (2000). The kinetics of CD154 (CD40L) expression in peripheral blood mononuclear cells of healthy subjects in liver allograft recipients and X-linked hyper-IgM syndrome.. Clin Transplant.

[25] Karin M, Ben-Neriah Y (2000). Phosphorylation meets ubiquitination: the control of NF-[kappa]B activity.. Annu Rev Immunol.

[26] Manis JP, Tian M, Alt FW (2002). Mechanism and control of class-switch recombination.. Trends Immunol.

[27] Pido-Lopez J, Whittall T, Wang Y, Bergmeier LA, Babaahmady K (2007). Stimulation of Cell Surface CCR5 and CD40 Molecules by Their Ligands or by HSP70 Up-Regulates APOBEC3G Expression in CD4+ T Cells and Dendritic Cells.. J Immunol.

[28] Pido-Lopez J, Wang Y, Seidl T, Babaahmady K, Vaughan R (2009). The effect of allogeneic in vitro stimulation and in vivo immunization on memory CD4(+) T-cell APOBEC3G expression and HIV-1 infectivity.. Eur J Immunol.

[29] Morner A, Jansson M, Bunnik EM, Scholler J, Vaughan R (2011). Immunization with recombinant HLA classes I and II, HIV-1 gp140, and SIV p27 elicits protection against heterologous SHIV infection in rhesus macaques.. J Virol.

[30] Harris RS, Liddament MT (2004). Retroviral restriction by APOBEC proteins.. Nat Rev Immunol.

[31] Wang Y, Seidl T, Whittall T, Babaahmady K, Lehner T (2010). Stress-activated dendritic cells interact with CD4+ T cells to elicit homeostatic memory.. Eur J Immunol.

[32] Saunderson SC, Schuberth PC, Dunn AC, Miller L, Hock BD (2008). Induction of exosome release in primary B cells stimulated via CD40 and the IL-4 receptor.. J Immunol.

[33] McLellan AD (2009). Exosome release by primary B cells.. Crit Rev Immunol.

[34] Khatua AK, Taylor HE, Hildreth JE, Popik W (2009). Exosomes packaging APOBEC3G confer human immunodeficiency virus resistance to recipient cells.. J Virol.

[35] Xu W, Santini PA, Sullivan JS, He B, Shan M (2009). HIV-1 evades virus-specific IgG2 and IgA responses by targeting systemic and intestinal B cells via long-range intercellular conduits.. Nat Immunol.

[36] Seidl T WT, Babaahmady K, Lehner T (2011). B cell agonists upregulate AID and APOBEC3G deaminases, which induce IgA and IgG class antibodies and anti-viral infection..

[37] Honjo T, Muramatsu M, Fagarasan S (2004). AID: how does it aid antibody diversity?. Immunity.

[38] Muramatsu M, Sankaranand VS, Anant S, Sugai M, Kinoshita K (1999). Specific expression of activation-induced cytidine deaminase (AID), a novel member of the RNA-editing deaminase family in germinal center B cells.. J Biol Chem.

[39] Neuberger MS, Harris RS, Di Noia J, Petersen-Mahrt SK (2003). Immunity through DNA deamination.. Trends Biochem Sci.

[40] Petersen-Mahrt SK, Harris RS, Neuberger MS (2002). AID mutates E. coli suggesting a DNA deamination mechanism for antibody diversification.. Nature.

[41] Santiago ML, Montano M, Benitez R, Messer RJ, Yonemoto W (2008). Apobec3 encodes Rfv3, a gene influencing neutralizing antibody control of retrovirus infection.. Science.

[42] Sprent J, Surh CD (2011). Normal T cell homeostasis: the conversion of naive cells into memory-phenotype cells.. Nat Immunol.

[43] Bevan MJ (2011). Understand memory, design better vaccines.. Nat Immunol.

[44] Babaahmady K, Oehlmann W, Singh M, Lehner T (2007). Inhibition of HIV-1 Infection of Human CD4+ T Cells by Microbial HSP70 and the Peptide Epitope 407**–**426..

[45] Sugiyama R, Nishitsuji H, Furukawa A, Katahira M, Habu Y (2011). Heat shock protein 70 inhibits HIV-1 Vif-mediated ubiquitination and degradation of APOBEC3G.. J Biol Chem.

[46] Huber M, Trkola A (2007). Humoral immunity to HIV-1: neutralization and beyond.. J Intern Med.

[47] Forthal DN, Moog C (2009). Fc receptor-mediated antiviral antibodies.. Curr Opin HIV AIDS.

[48] Kim EY, Bhattacharya T, Kunstman K, Swantek P, Koning FA (2010). Human APOBEC3G-mediated editing can promote HIV-1 sequence diversification and accelerate adaptation to selective pressure.. J Virol.

[49] Sadler HA, Stenglein MD, Harris RS, Mansky LM (2010). APOBEC3G contributes to HIV-1 variation through sublethal mutagenesis.. J Virol.

[50] Scholler J, Singh M, Bergmeier L, Brunstedt K, Wang Y (2010). A recombinant human HLA-class I antigen linked to dextran elicits innate and adaptive immune responses.. J Immunol Methods.

[51] Gilbert P, Wang M, Wrin T, Petropoulos C, Gurwith M (2010). Magnitude and breadth of a nonprotective neutralizing antibody response in an efficacy trial of a candidate HIV-1 gp120 vaccine.. J Infect Dis.

