# Supplementary figures and images for: The Role of Innate APOBEC3G and Adaptive AID Immune Responses in HLA-HIV/SIV Immunized SHIV Infected Macaques
Source: PLoS One. 2012 Apr 13;7(4):e34433. doi: 10.1371/journal.pone.0034433 (PMC3326050; doi:10.1371/journal.pone.0034433)

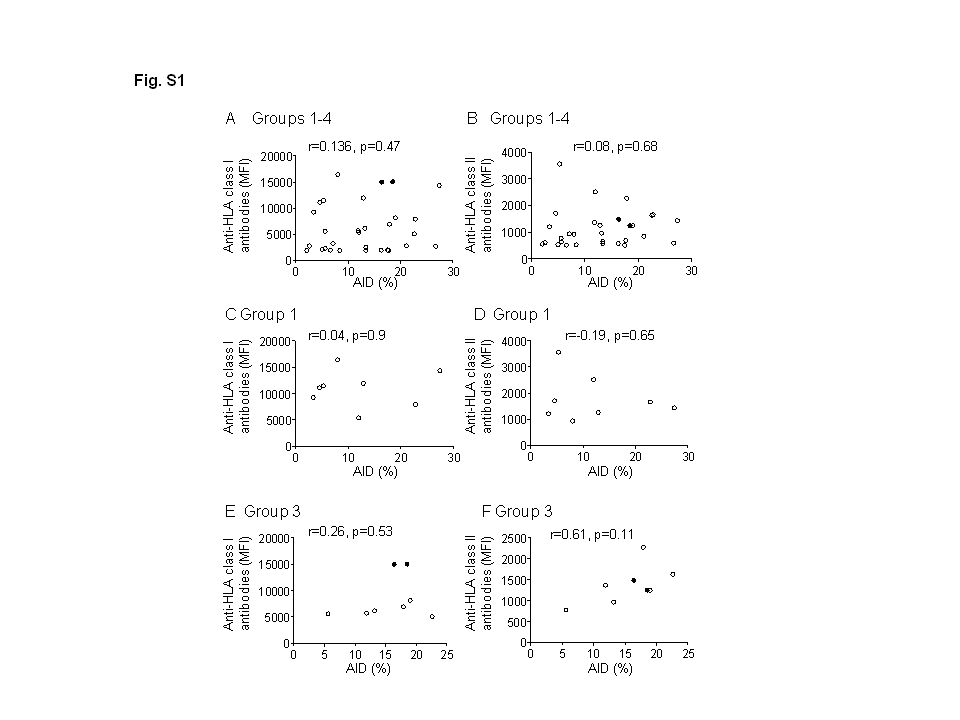

Supplement: Figure S1 — Correlation between AID and HLA-class I antibodies in groups 1–4, 1 and 3. Correlation between AID in CD20+ B cells and anti-HLA class I (A,C,E) or anti-HLA class II antibodies (B, D, F); (A,B) in the immunized groups 1–4, (C,D) in group 1 and (E,F) in group 3 macaques. In all figures the 2 uninfected macaques in group 3 are indicated by a solid circle. (TIF) [file pone.0034433.s001.tif]

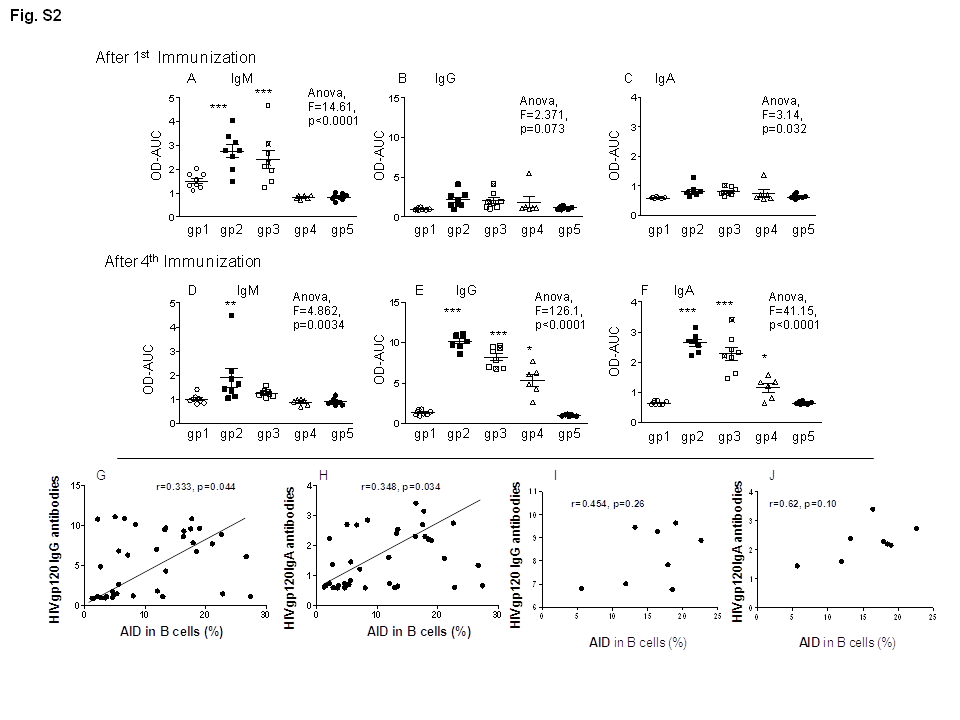

Supplement: Figure S2 — Comparison of HIVgp120 antibodies and correlation with AID in B cells. Comparison of serum HIV gp120 specific IgM, IgG and IgA antibodies, in the 5 groups of macaques after the 1st (A–C) and 4th (D–F) immunization; correlation between AID in CD20+ B cells and HIVgp120 IgG (G) or IgA (H) antibodies in all groups and in group 3 macaques (I,J). The antibodies were measured by ELISA and expressed as mean (±sem) of the OD (area under the curve). *p<0.05, **p<0.01 and ***p<0.001 compared with the untreated group 5 controls. In all figures the 2 uninfected macaques in group 3 are indicated by a solid circle. (TIF) [file pone.0034433.s002.tif]

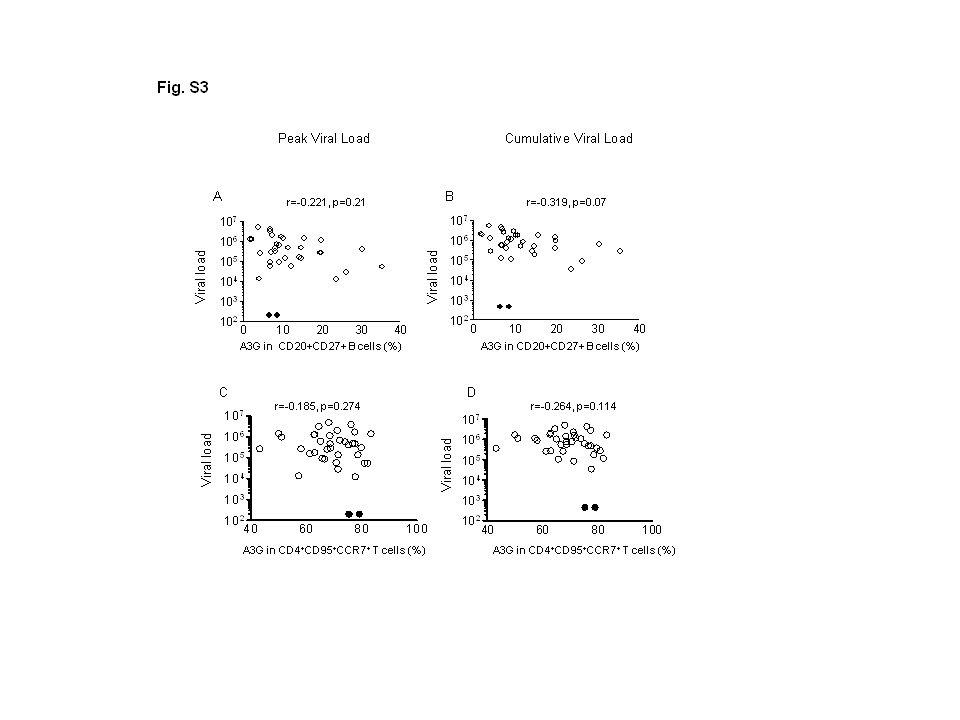

Supplement: Figure S3 — Indirect trend of correlation between the viral load and A3G in memory B and T cells. Indirect trend of correlation between the peak or cumulative viral load and A3G in (A,B) CD20+CD27+ memory B cells and (C,D) CD4+CD95+CCR7+ memory T cells. In all figures the 2 uninfected macaques in group 3 are indicated by a solid circle. (TIF) [file pone.0034433.s003.tif]

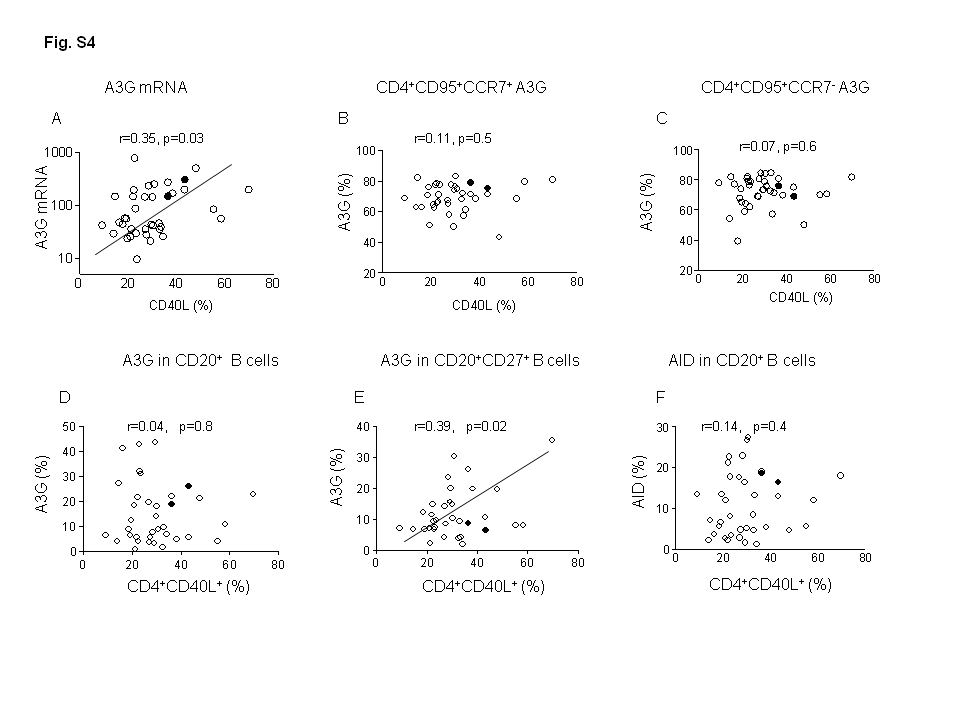

Supplement: Figure S4 — Correlation between CD40L and A3G mRNA in PBMC or A3G in memory B and central T cells. Correlation between CD4+ CD40L+ T cells and (A) A3G mRNA in PBMC, (B) A3G protein in CD4+CD95+CCR7+ central, (C) CD4+CD95+CCR7- effector memory T cells, (D) CD20+ B cells and (E) CD20+CD27+ memory B cells. (F) Correlation between CD40L and AID in CD20+ B cells in the 5 groups of animals. In all figures the 2 uninfected macaques in group 3 are indicated by a solid circle. (TIF) [file pone.0034433.s004.tif]

## Slide 1
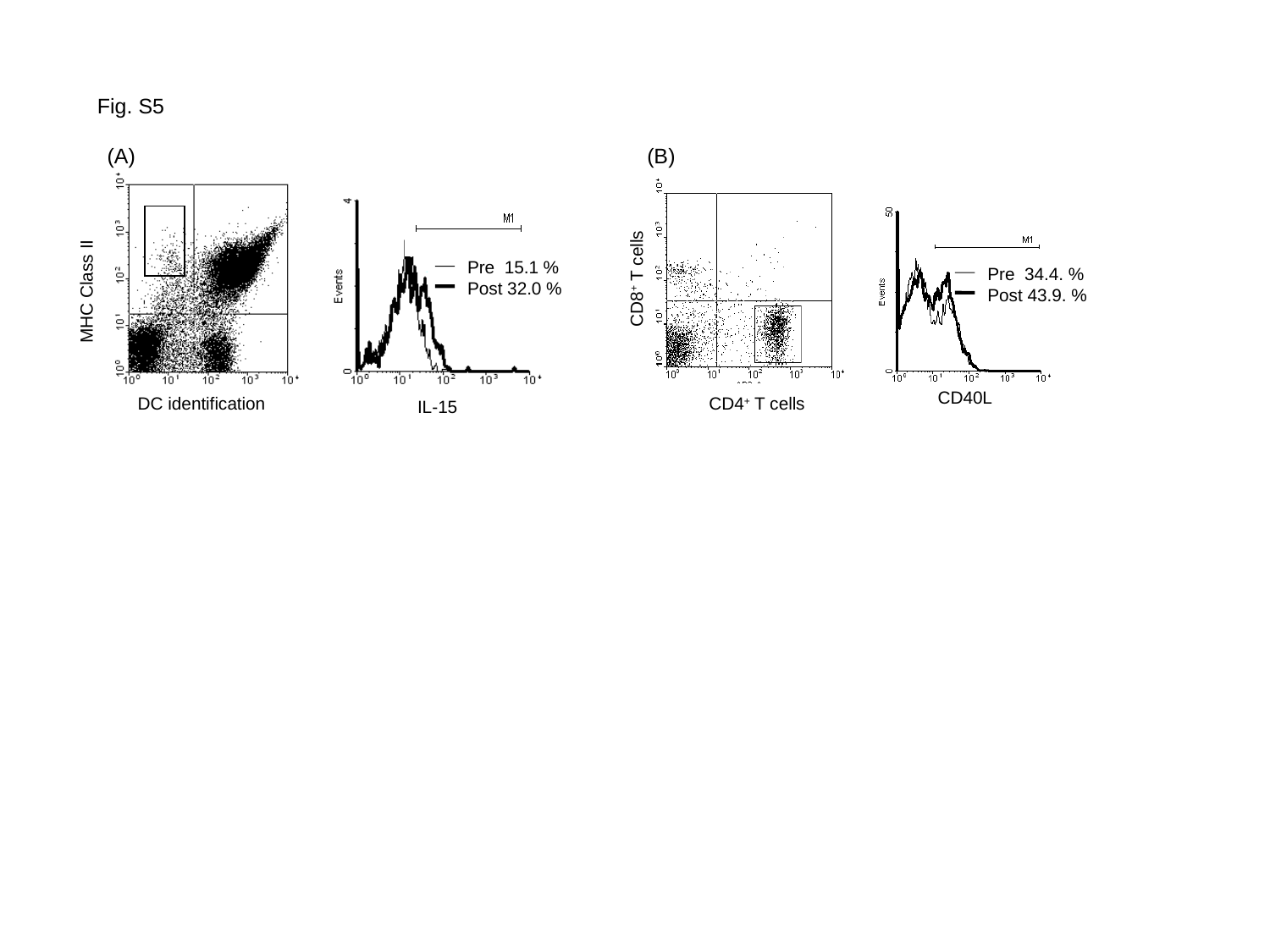

Fig. S5
(A)
(B)
Pre 15.1 %
Post 32.0 %
Pre 34.4. %
Post 43.9. %
CD8+ T cells
MHC Class II
CD40L
DC identification
CD4+ T cells
IL-15

Supplement: Figure S5 — Rrepresentative flow cytometry of maIL-15 DC and CD40L expression of CD4+ T cells. Representative flow cytometry illustrations are presented (A) for maIL-15 and (B) CD40L; pre- (thin line) and post-immunization (bold line). In all figures the 2 uninfected macaques in group 3 are indicated by a solid circle. (PPT) [file pone.0034433.s005.ppt]
